# Danger of Delay: A Case Report of a Hidden, Extensive, Congenital External Auditory Canal Cholesteatoma in a Pediatric Patient

**DOI:** 10.7759/cureus.81938

**Published:** 2025-04-09

**Authors:** Stacy Arvinna Binti Jamarun, Cheng Ai Ong, Elsie J Anastasius, Bee-See Goh

**Affiliations:** 1 Department of Otorhinolaryngology - Head and Neck Surgery, Hospital Queen Elizabeth, Kota Kinabalu, MYS; 2 Department of Otorhinolaryngology - Head and Neck Surgery, National University of Malaysia, Kuala Lumpur, MYS

**Keywords:** canal cholesteatoma, cholesteatoma, congenital cholesteatoma, external auditory canal cholesteatoma, hidden cholesteatoma, pediatric cholesteatoma

## Abstract

Congenital aural stenosis (CAS) is a rare congenital ear abnormality affecting the external and middle ear. It has a higher risk of developing external auditory canal cholesteatoma (EACC) compared to congenital aural atresia. CAS is defined when the external auditory canal has a diameter of 4 mm or less. This size makes it difficult to examine and confirm the diagnosis.Thus, a computed tomography (CT) scan is necessary to be performed despite the absence of clinical symptoms indicative of cholesteatoma in CAS patients. The findings from the CT scan can guide further management. This is a case of extensive EACC complicated by a brain abscess in a young boy with CAS. Due to the patient factors, a timely CT scan to detect EACC was impeded, which resulted in more serious complications. Our case report aims to underscore the peril of postponing appropriate management of this disease.

## Introduction

External auditory canal cholesteatoma (EACC) is a condition where a cholesteatoma (an abnormal growth of skin cells) forms in the external auditory canal (EAC) at birth or during early childhood. EACC was initially described in 1850 and further distinguished from keratosis obturans in 1980. However, classification of EACC based on its histology and clinical symptoms was only introduced in 2005 [1]. EACC patients commonly present with conductive hearing loss, foul-smelling otorrhea, and otalgia, with or without vertigo, whereas clinical features include bone erosion, squamous epithelium invasion, localized periostitis, and bone sequestration [2]. It is frequently linked to congenital aural stenosis (CAS), a condition where the ear canal is abnormally narrow or obstructed at birth, as cholesteatoma occurs in 48% of CAS cases [3]. Thus, even if there are no clinical signs indicating cholesteatoma, a computed tomography (CT) scan is occasionally performed on a CAS patient over the age of four years. We report a case of extensive EACC complicated by brain abscess in a young boy with CAS.

## Case presentation

An eight-year-old boy was presented to the emergency department due to fever, severe frontal headache, neck stiffness, occasional vomiting, and reduced hearing in the left ear. He had a history of admission to the ward for left mastoiditis at the age of one year and was diagnosed to have congenital left ear canal stenosis. Unfortunately, the patient defaulted on follow-up without having any hearing assessment or radiological imaging done. At five years old, he claimed to have left postauricular swelling where an incision and drainage were performed at a private healthcare center, but was not referred to any otorhinolaryngologist.

General examination showed that the patient had an intact neurological examination with no photophobia; however, he had right neck torticollis with limited neck movement. There was a left posterior auricular swelling measuring 3 x 2 cm, pushing the pinna forward. Swelling was firm, tender, and warm with no fluctuant area. Otoscopy findings showed left canal stenosis with a small opening (pin-hole), which could not be examined with the otoscope, while the right ear canal was dry with central perforation of the tympanic membrane. Hearing assessment could not be performed due to the patient being in severe pain.

**Figure 1:**
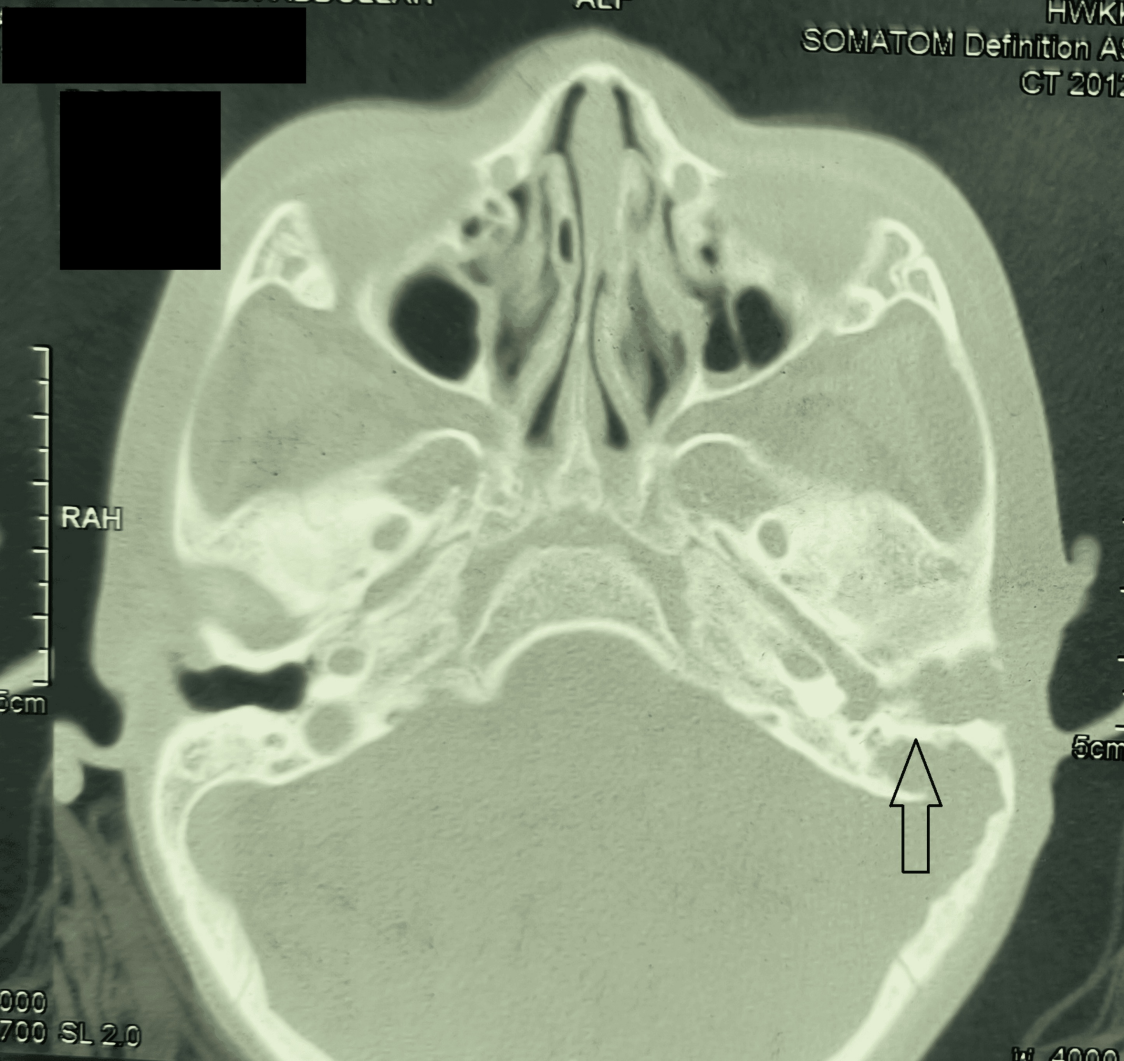
CT in axial section of the temporal bone. It showed a heterogeneous enhancing lesion seen within the left side of the ear canal and mastoid cavity. The ossicles were not visualized, and the scutum was eroded. The lateral semicircular canal appeared to have a dehiscence as well. Posteriorly, there was a rim enhancement at the left cerebellar region measuring 2.2 (anteroposterior) x 1.8 (width) x 1.8 (craniocaudal). No evidence of hydrocephalus was present, but there was a mass effect causing compression to the adjacent sigmoid and transverse sinus; however, it was preserved. The facial nerve canal was intact.

The patient’s C-reactive protein was raised alongside leucocytosis. A contrasted CT of the brain and high-resolution CT temporal bone was performed, and it showed a heterogenous enhancing lesion within the left ear canal and mastoid cavity. The ossicles were not visualized, and the scutum was eroded. The lateral semicircular canal appeared to have a dehiscence as well. Posteriorly, there was a rim enhancement at the left cerebellar region measuring 2.2 (anteroposterior) x 1.8 (width) x 1.8 (craniocaudal) cm. Evidence of hydrocephalus was absent, but there was a mass effect causing compression to the adjacent sigmoid and transverse sinus; however, it was preserved. The facial nerve canal was intact (Figure 1).

**Figure 2:**
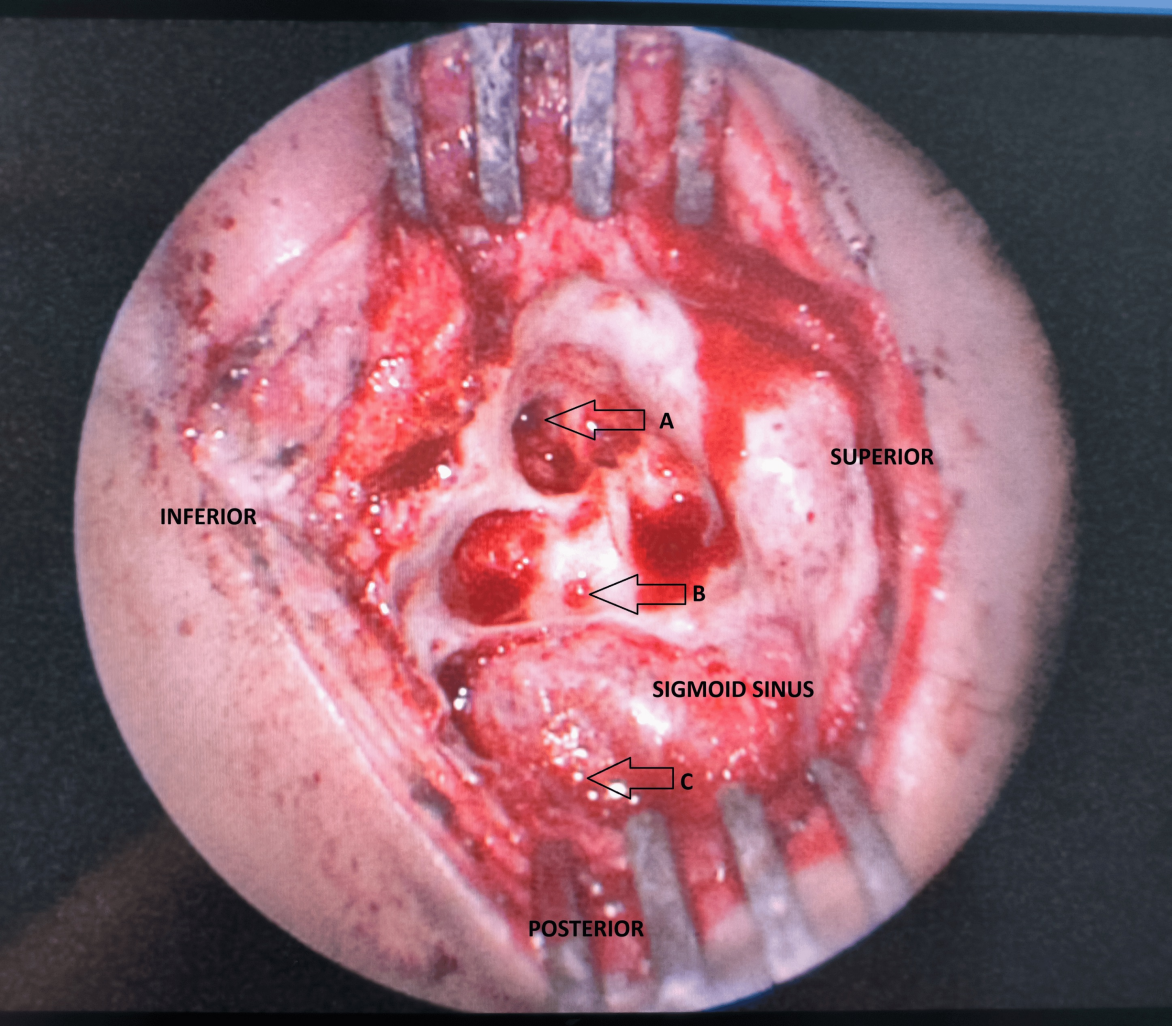
These are the intraoperative findings of this case. The left external ear canal (arrow labelled A) was identified. The canal has a stenosed opening and a bony defect at the posterior part of the wall. There is another bony defect at the cortex of the mastoid cavity (arrow labelled B). Copious pus was discharging through this defect. Slightly posterior to the defect (arrow labelled C), the dura was exposed alongside the sigmoid sinus.

The boy was treated with a left radical mastoidectomy. Intraoperative findings showed the left ear canal was stenosed with an opening measuring approximately 3 mm and the presence of a bony defect at the posterior part of the canal. Cholesteatoma was filling up the entire left ear canal, middle ear cavity, and mastoid cavity. It extended posteriorly until the mastoid tip and anteriorly into the eustachian tube opening. Ossicles were eroded. Cholesteatoma was removed completely. The facial nerve was identified and intact despite revealing an exposed structure near the second genu, at the posterior part of the tympanic segment. There was another small bony defect at the cortex of the mastoid bone with some pus discharging, and another part where the dura exposed together with the sigmoid sinus. However, dura was intact, and no cerebrospinal fluid leak was seen (Figure 2).

Postoperatively, the patient's neck stiffness and headache had resolved. Repeated contrast-enhanced CT of the brain at 2nd and 4th weeks postoperatively showed the resolution of the left cerebellar abscess.

## Discussion

CAS is defined as the EAC with a diameter of 4 mm or less [3]. When compared to congenital aural atresia, CAS carries a substantially higher risk of cholesteatoma, particularly in patients with a bony ear canal aperture of 2 mm or less. The canal architecture in CAS makes it more likely for squamous debris to become trapped and lead to the development of EACC. Bony erosion, squamous epithelium invasion, localized periostitis, and bone sequestration are characteristics of EACC. Compared to middle ear cholesteatoma, the EACC is 60 times less common [2]. Lesion commonly affects the floor of the EAC, and differential diagnosis includes skull base osteomyelitis and squamous cell carcinoma. Even though the EACC diagnosis is based on clinical findings, typically a CT image will demonstrate involvement of the middle ear and mastoid bone [2,4]. It was recommended to perform a computed tomography of the temporal bone after four years old to look out for a hidden canal cholesteatoma, and it would provide a good assessment tool for surgical repair intention. In fact, Jahrsdoerfer et al. developed a 10-point grading system to determine surgical candidacy based on key features from the CT scan and the appearance of the external ear [5]. Shin et al. have introduced another staging system based on CT scan to provide guidance in deciding the surgery type more suitable for the patient [6].

Some cases of acute mastoiditis in children were noted to have underlying congenital or acquired cholesteatoma [2]. In that case, acute infection should be managed with intravenous antibiotics conservatively. Then, if indicated, a surgery limited to achieving drainage and reducing the volume of cholesteatoma, especially in cases with encapsulated abscesses or abscesses of the cerebellum, should be performed [2,7]. A definitive cholesteatoma surgery should be performed a few weeks after the acute infection treatment.

Such as in our case, upon presentation with acute mastoiditis accompanied by CAS, a CT of the brain and a high-resolution temporal CT should be promptly arranged. This decisive radiological imaging shows that this patient has an extensive cholesteatoma with evidence of collection in the cerebellar region. For him, surgical intervention is imminent. The surgical intention was not to maintain hearing but to evacuate the source of infection while creating a patent ear canal with preservation of skin but less risk of trapping squamous debris [6].

## Conclusions

CAS is frequently associated with EACC. Early detection of EACC is crucial because it helps in preventing potential complications that could arise from untreated cholesteatoma, such as hearing loss and infection spread. The clinical findings of this patient demonstrate the value of CT in the diagnosis of EACC, particularly in those with CAS. We would like to emphasize the need for early detection by continuous monitoring, meticulous diagnostic evaluation, and personalized surgical management for patients with CAS and EACC, particularly in young children.
